# Supplementary material for: Characterization of Persistent Uncontrolled Asthma Symptoms in Community Members Exposed to World Trade Center Dust and Fumes
Source: Int J Environ Res Public Health. 2020 Sep 11;17(18):6645. doi: 10.3390/ijerph17186645 (PMC7558705; doi:10.3390/ijerph17186645)
Supplement: Supplementary file 1 [file ijerph-17-06645-s001.pdf]

Supplemental Tables

Table S1. Demographic, clinical and exposure characteristics of total study patients (n = 73).

	Level	Total	Uncontrolled	Controlled	P
**N**		**73**	**54**	**19**	
**Sex, n (%)**	*F*	51 (70)	36 (67)	15 (79)	0.39
	M	22 (30)	18 (33)	4 (21)	
**Age, median [IQR]**		57 [50,62]	56.5 [48,62]	58 [51,63]	0.62
**Race/Ethnicity, n (%)**	*Hispanic*	33 (45)	28 (52)	5 (26)	0.12
	NH-White	15 (21)	10 (19)	5 (26)	
	NH-Black	20 (27)	24 (26)	6 (32)	
	Asian or other	5 (7)	2 (4)	3 (16)	
**BMI median [IQR]**		29.4 [26.5,32.9]	29.4 [27,32.9]	29.4 [26,34]	0.96
**BMI, n (%)**	*Normal (<25)*	8 (11)	4 (7)	4 (21)	0.29
	Overweight (25-30)	33 (45)	26 (48)	7 (37)	
	Obese (≥30)	32 (44)	24 (44)	8 (42)	
**Dust cloud, n (%)**	*No*	36 (49)	25 (46)	11 (58)	0.43
	Yes	37 (51)	29 (54)	8 (42)	
**Exposure category, n (%)**	*Clean-up Worker*	16 (22)	12 (22)	4 (21)	0.59
	Other	7 (10)	4 (7)	3 (16	
	Resident	9 (12)	8 (15)	1 (5)	
	Local Worker	41 (56)	30 (56)	11 (58)	
**ASUI Score, median [IQR]**		0.66 [0.43, 0.70]	0.59 [0.43, 0.70]	0.82 [0.71,0.89]	0.001
**MMRC, n (%)**	*(≤3)*		44 (81)	17 (90)	0.72
	(>3)		10 (19)	2 (10)	
**6MWT, Median Distance [IQR]**		401 [349.8,454.3]	396 [346.3,429.2]	419.6 [365.8, 462.0]	0.29

Table S2. Upper airway symptoms in total study group (N=73).

	Total	Uncontrolled	Controlled	P value
**ICSD median [IQR]**	30.5 [18.3,38.8]	35 [22.8, 48]	25 [14.3, 29.5]	0.005
**LCQ, median [IQR]**	13.9 [11.2,16.8]	13 [9.7, 16.3]	16.2 [12.6, 20]	0.02
**VHI, median [IQR]**	4.5 [0.0,15.3]	6.5 [0.3, 16]	0.0 [0.0, 9.8]	0.08

Table S3. Spirometry/FOT measures in total participants (n = 73).

	Level	Total	Uncontrolled	Controlled	P-value for % pred
		**SPIROMETRY**			
**Pre BD**					
**FVC,L (%pred, median)**		3.2 (96.5)	3.1 (94.5)	3.3 (98.5)	0.93
**FEV_1,L (%pred, median)**		2.5 (92.5)	2.5 (90.5)	2.3 (96.5)	0.21
**FEV_1/FVC, median**		78.3 [73.5,82.4]	78.0 [72.7,82.3]	80 [75.3,82.6]	0.26
**Post BD**					
**FVC,L (%pred, median)**		3.1 (98.0)	3.1 (98.0)	3.1 (100)	0.98
**FEV_1,L (%pred, median)**		2.5 (96.0)	2.5 (95.0)	2.4 (96.5)	0.81
**FEV_1/FVC, median**		78.3 [75.0,81.8]	78.3 [74.6,81.8]	78.5 [75.4,81.7]	0.69
		**FOT Measurements**			
**Pre BD**					
**R5 median [IQR]**		4.5 [3.4,5.7]	4.4 [3.5,5.7]	4.7 [3.4, 5.7]	0.97
**R5-20 median [IQR]**		1.0 [0.5,1.5]	1.0 [0.5,1.5]	0.8 [0.4, 1.4]	0.58
**Post BD**					
**R5 median [IQR]**		4.2 [3.4,5.3[	4.2 [3.5,5.3]	4.4 [3.3, 5.7]	1.0
**R5-20 median [IQR]**		0.8 [0.4,1.3]	0.9 [0.5,1.3]	0.6 [0.4, 1.2]	0.43

Table S3a. Spirometry/FOT measures and airway hyperresponsiveness status comparing those with ACT < 15 (n = 29) and > 20, n = 19).

	Level	Total	Uncontrolled	Controlled	P-value for % pred
		**SPIROMETRY**			
**Pre BD**					
**FVC,L (%pred, median)**		3.0 (95)	3.0 (94)	3.3 (98.5)	0.90
**FEV_1,L (%pred, median)**		2.4 (95)	2.4 (90)	2.3 (96.5)	0.29
**FEV_1/FVC, median**		78.5 [73.6, 82.6]	78.5 [71.7, 82.4]	80 [75.3,82.6]	0.26
**Post BD**					
**FVC,L (%pred, median)**		3.1 (99)	3.1 (98.5)	3.13 (100)	0.79
**FEV_1,L (%pred, median)**		2.4 (96)	2.5 (95.5)	2.4 (96.5)	0.88
**FEV_1/FVC, median**		79.4 [75.2, 82.6]	79.4 [75.2, 82.8]	78.5 [75.4,81.7]	0.86
**FOT Measurements**					
**Pre BD**					
**R5 median [IQR]**		4.2 [3.3, 5.6]	4.2 [3.3,5.5]	4.7 [3.4, 5.7]	0.61
**R5-20 median [IQR]**		0.6 [0.3, 1.5]	0.6 [0.3,1.5]	0.8 [0.4, 1.4]	0.80
**Post BD**					

**R5 median [IQR]**		4.2 [3.3, 5.5]	4.1 [3.3,4.8]	4.4 [3.3, 5.7]	0.74
**R5-20 median [IQR]**		0.6 [0.4, 1.1]	0.6 [0.4,1.1]	0.6 [0.4, 1.2]	0.82
**Bronchial Hyper-responsiveness (row-%)**					
**BHR at dose ≤16mg (%)**	*Yes*	25	14 (56%)	11 (44%)	0.51
	No	17	12 (70.6%)	5 (29.4%)	
	NA	6			

Table S4. Airway responsiveness measured by BHR and PVFM in total participants (N=73)

	Level	Total	n (% Controlled)	P value
**BHR (at dose ≤16mg)**	*Negative*	27	5 (18.5%)	0.24*
	Positive	32	11 (34.4%)	
	NA	14		
**Any PVFM n (%)**	*Negative*	33	7 (22.6%)	0.41*
	Positive	34	11 (32.4%)	
	NA	6		
**PVFM unprovoked, n (%)**	*Negative*	59	15 (25.4%)	0.21*
	Positive	8	4 (50%)	
	NA	6		
**PVFM provoked, n (%)**	*Negative*	34	8 (23.5%)	0.59*
	Positive	34	11 (32.4%)	
	NA			
**PVFM and BHR, n (%)**	*-PVFM, -BHR*	9	0 (0.0%)	0.0002**
	-PVFM, +BHR	18	5 (27.8%)	
	+PVFM, -BHR	17	5 (29.4%)	
	+PVFM,+BHR	13	6 (46.2%)	
	NA	16		

*Fisher exact test; ** Exact Cochran-Amitage trend test (catt_exact).
